# SOX30 is required for male fertility in mice

**DOI:** 10.1038/s41598-017-17854-5

**Published:** 2017-12-15

**Authors:** Chun-Wei Allen Feng, Cassy Spiller, Donna J. Merriner, Moira K. O’Bryan, Josephine Bowles, Peter Koopman

**Affiliations:** 1 School of Biomedical Sciences, The University of Queensland, Queensland, Australia; 2 Institute for Molecular Bioscience, The University of Queensland, Queensland, Australia; 3 School of Biological Sciences, Monash University, Victoria, Australia

## Abstract

Male infertility is a major and growing problem and, in most cases, the specific root cause is unknown. Here we show that the transcription factor SOX30 plays a critical role in mouse spermatogenesis. *Sox30*-null mice are healthy and females are fertile, but males are sterile. In the absence of *Sox30* meiosis initiates normally in both sexes but, in males, germ cell development arrests during the post-meiotic round spermatid period. In the mutant testis, acrosome and axoneme development are aberrant, multinucleated germ cells (symplasts) form and round spermatids unable to process beyond step 3 of spermiogenesis. No elongated spermatids nor spermatozoa are produced. Thus, *Sox30* represents a rare example of a gene for which loss of function results in a complete arrest of spermatogenesis at the onset of spermiogenesis. Our results suggest that *SOX30* mutations may underlie some instances of unexplained non-obstructive azoospermia in humans.

## Introduction

Spermatogenesis, the process whereby diploid spermatogonia differentiate into mature haploid spermatozoa, is highly complex and involves many precisely coordinated steps. In the post-natal phase spermatogonia undergo several rounds of mitotic division to produce primary spermatocytes which, in turn, duplicate their DNA content then undergo two rounds of nuclear division (meiosis I and meiosis II) resulting in formation of haploid round spermatids. During the post-meiotic developmental phase, known as spermiogenesis, round spermatids undergo a complicated restructuring program that includes compaction of DNA, ejection of cytoplasm and formation of the acrosome and flagellum^1,2^. Finally, spermatids are released from the epithelium via a process known as spermiation. Several thousand genes are expressed during spermatogenesis, although the function of the majority is unknown^3–5^.

In the mouse, spermiogenesis is divided into four phases characterized by the presence of round spermatids, elongating spermatids, condensing spermatids and condensed spermatids. In the elongating and condensing spermatids there is dramatic remodelling of chromatin structure that involves the sequential replacement of histones with transition nuclear proteins (TNPs) and eventually by protamines (PRMs), both of which are germ cell specific chromatin proteins. Transcription is most active in late spermatocytes and round spermatids^6^; this is the time point when *Crem* (cAMP-responsive element modulator), which encodes a key transcriptional activator CREMτ (CREM-tau), is expressed at highest levels^7,8^. Subsequently, transcription ceases during the spermatid elongation phase because PRMs compact the DNA into an inactive state.

Members of the *SOX* (*S* *ry*-related High Motility Group (HMG) box) gene family encode transcription factors that are highly conserved and are important for a range of developmental processes, including sex determination, neuronal development and regulation of stem cell pluripotency^9^. Within the SOX family SOX30 is the sole representative of SOX group H and is a relatively divergent member, with an HMG domain only 46% identical to the SOX-HMG box consensus sequence^10,11^. Apart from the HMG domain, SOX30 has no significant homology to any known protein functional domains. Perhaps due to its singular classification and lack of other known domains, *Sox30* has remained under-characterised to date. In mice, it has been reported that the expression of *Sox30* is specific to adult male germ cells and that the timing of expression correlates to spermatocytes undergoing meiosis^11^. A recent analysis of publicly-available RNA and protein profiling and interaction network data predicted a role for *Sox30* in spermatogenesis in both humans and mouse^12^.

Here we describe the expression pattern of *Sox30* in mouse fetal ovary and pubertal testis demonstrating that, in both sexes, *Sox30* expression begins in germ cells shortly after expression of the pre-meiotic marker, *Stra8*. This expression pattern suggested that SOX30 might be essential for successful meiosis in both sexes but, counter to this, we observe no meiotic abnormality in either sex in a *Sox30*-null mouse model. Instead, our results indicate that SOX30 is a key transcription factor necessary for normal progression of spermatogenesis at the post-meiotic spermiogenesis phase. The clear-cut infertility phenotype we reveal, including a complete lack of elongated spermatids, demonstrates that this protein is essential for male fertility in mice.

## Results

### Expression of *Sox30* follows that of *Stra8* in both fetal ovary and postnatal testis

**Figure 1:**
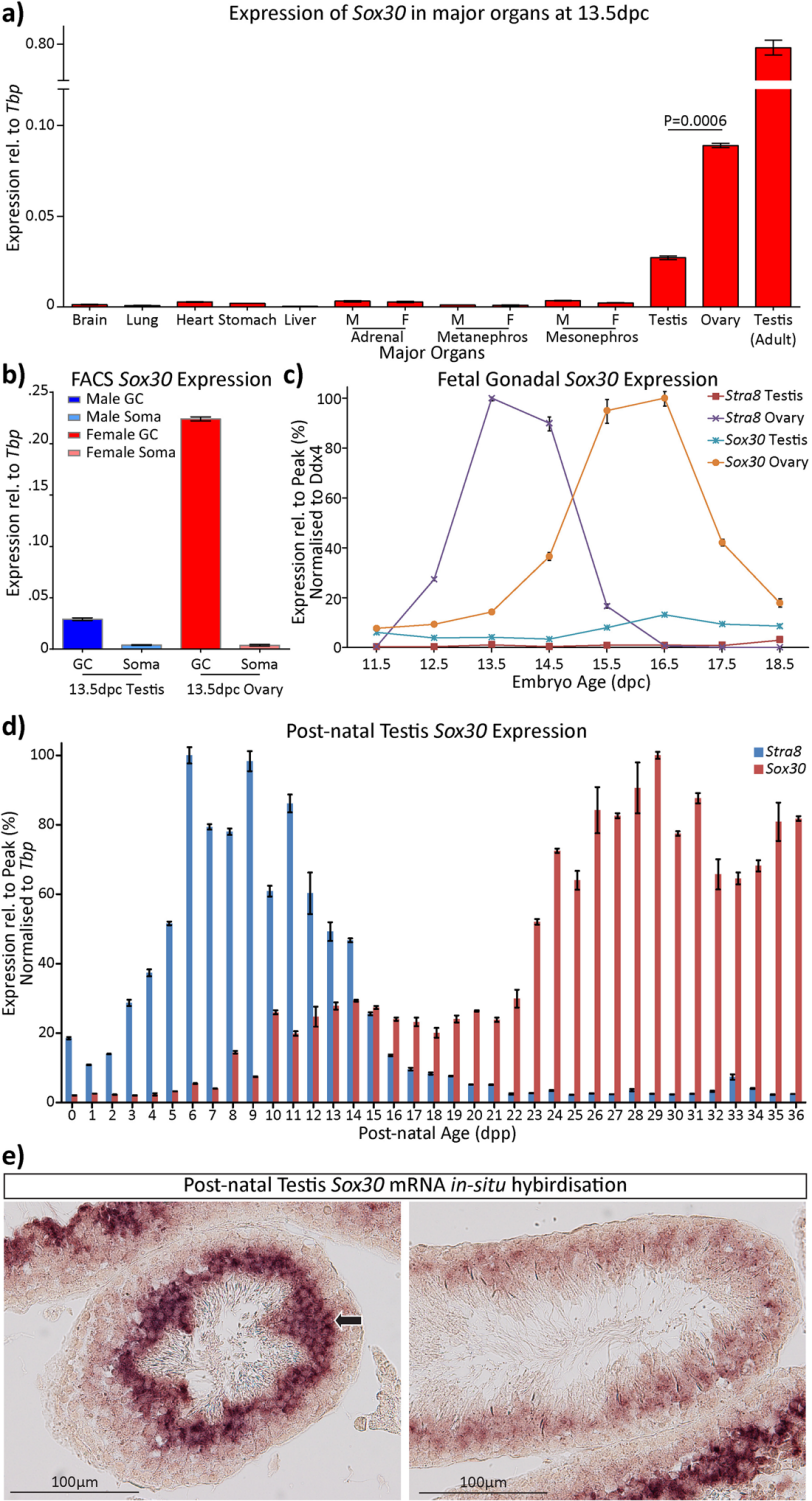
Expression of *Sox30* is specific to the germ cells of the gonads at 13.5 dpc and follows the expression of *Stra8* in both fetal ovaries and postnatal testis. (**a**) qRT-PCR was performed on various tissues of 13.5 dpc embryos, and adult testis, to assess *Sox30* expression. Robust *Sox30* expression in embryonic tissue is observed only in the gonads, with significantly higher levels detected in the fetal ovary than the fetal testis. *Sox30* expression is ~8 fold higher in the adult testis than in the fetal ovary (pools of 4~8 individuals per column; two-tailed unpaired t-test). (**b**) qRT-PCR analysis of flow-sorted (FACS) populations of fetal gonadal tissues reveals that *Sox30* transcript is enriched in the germ cell compartment in both the fetal ovary and testis at 13.5 dpc (cells isolated from pools of 4 to 8 gonad pairs). (**c**) qRT-PCR analysis in fetal gonads (from 11.5 to 18.5 dpc) demonstrates that the expression of *Sox30* in the fetal ovary increases after the expression of *Stra8* (pools of 4 to 8 gonad pairs). (**d**) qRT-PCR analysis demonstrates that expression of *Sox30* follows expression of *Stra8* in the postnatal testis (each time point represents one individual sample). All error bars represent S.E.M. of technical replicates. *Ddx4* is expressed specifically in germ cells and is used as a normalizer for germ cell-specific genes when whole fetal gonadal tissue is analysed. (**e**) We verified by mRNA *in situ* hybridisation the previously described germ cell specific expression of *Sox30* in the post-natal testis^11^. Transcripts for *Sox30* were detected beginning at mid-pachytema (right panel), peaking in round and elongating spermatids (left panel, arrow). Elongated spermatids have diminished expression and no signal was detected in sperm just prior to spermiation.

Previously we conducted an Affymetrix microarray analysis using fetal gonadal tissue of both sexes and at several time points^13^ and found that the *Sox30* transcript was more highly expressed in the developing ovary than in developing testis (data not shown). At 13.5 dpc, we found by qRT-PCR that *Sox30* was expressed in the fetal gonads, with significantly higher levels in ovaries than testes (Fig. 1a). There was very little expression in the other fetal organs tested, but high *Sox30* expression was observed in the adult testis, as noted previously^11^. We purified fetal germ cells from the Oct4ΔPE:eGFP mouse line^14^, using FACS, and found that the expression of *Sox30* in both the fetal ovaries and testis was exclusive to germ cells (Fig. 1b).

The increased abundance of *Sox30* mRNA in the fetal ovary coincided with the peak expression of pre-meiotic marker *Stra8*. *Sox30* expression was highest at 15.5 to 16.5 dpc, just after *Stra8* is downregulated (Fig. 1c). Levels of *Sox30* expression remained low during fetal testicular development. In the postnatal testis *Sox30* mRNA became more abundant at around 8 dpp, which is coincident with the appearance of preleptotene spermatocytes, and continued to increase, peaking at approximately 29 dpp (Fig. 1d). In the early postnatal testis, *Sox30* mRNA was also first detected during the peak expression of *Stra8*, but highest *Sox30* mRNA abundance was observed after *Stra8* expression diminished. The fact that *Sox30* expression closely follows expression of the pre-meiotic marker *Stra8* ^15^ in both the fetal ovaries and postnatal testis led us to hypothesise that *Sox30* plays a role during germ cell meiosis in both sexes.

We attempted to visualize SOX30 protein in fetal ovaries and postnatal testis tissues but we were unsuccessful using commercially-available antibodies (See Supplementary Figure 1). *In lieu* of a suitable antibody, i*n-situ* hybridisation for *Sox30* mRNA was used to confirm the germ cell specific expression of *Sox30* in the post-natal testis and the pattern of its expression. *Sox30* mRNA was first detected during mid-pachynema before being dramatically up-regulated in round spermatids and elongating spermatids. Expression of *Sox30* mRNA was subsequently decreased in elongated spermatids and was not detected in sperm just prior to spermiation (Fig. 1e).

### *Sox30* deletion results in male infertility

We produced *Sox30*-heterozygous null mice using ES cells imported from KOMP (see Materials and Methods) and bred them to homozygosity. No gross phenotypic or behavioural abnormalities were observed. At 8 weeks of age, three male and two female homozygous *Sox30*-null mice were mated with wildtype C57BL/6 partners for test breeding. All males exhibited normal mating behaviour, and copulatory plugs were observed. However, over three months, none of the *Sox30-*null studs impregnated their partners. The females were replaced once during this period and the females removed from breeding were monitored for at least another 19 days for potential pregnancies. In contrast, *Sox30*-null females were normally fertile, disproving our initial hypothesis that SOX30 plays a critical role during meiosis in both sexes.

### Absence of Sox30 does not impact on oocyte development during fetal life

Although *Sox30* female mice were fertile, we found the transient expression of *Sox30* shortly after *Stra8* expression in fetal ovarian germ cells striking (Fig. 1c). We noted that SOX30 is also expressed in a female-specific manner during human fetal gonad development^16^. Therefore, we conducted further studies to determine if a subtle defect in meiotic progress in ovarian fetal germ cells could be identified. We checked for abnormalities in expression of meiosis-related genes in the *Sox30*-null fetal ovary. By qRT-PCR we saw lower than normal expression of *Stra8* in *Sox30-*null ovaries at 15.5 dpc but expression of later meiotic markers *Spo11*, *Sycp3*, *Dmc1* and *Rec8* remained relatively unaffected or was even slightly enhanced (Supplementary Figure 2a). Histological analysis by immunofluorescence for meiotic marker SYCP3 at 16.5 dpc and H&E staining did not highlight any significant abnormalities in *Sox30*-null fetal ovaries (Supplementary Figure 2b,c). After considering these results, and the fertility of *Sox30*-null female mice, we concluded that SOX30 is not required for female fertility.

### *Sox30* deletion led to spermatogenic arrest at round spermatids

**Figure 2:**
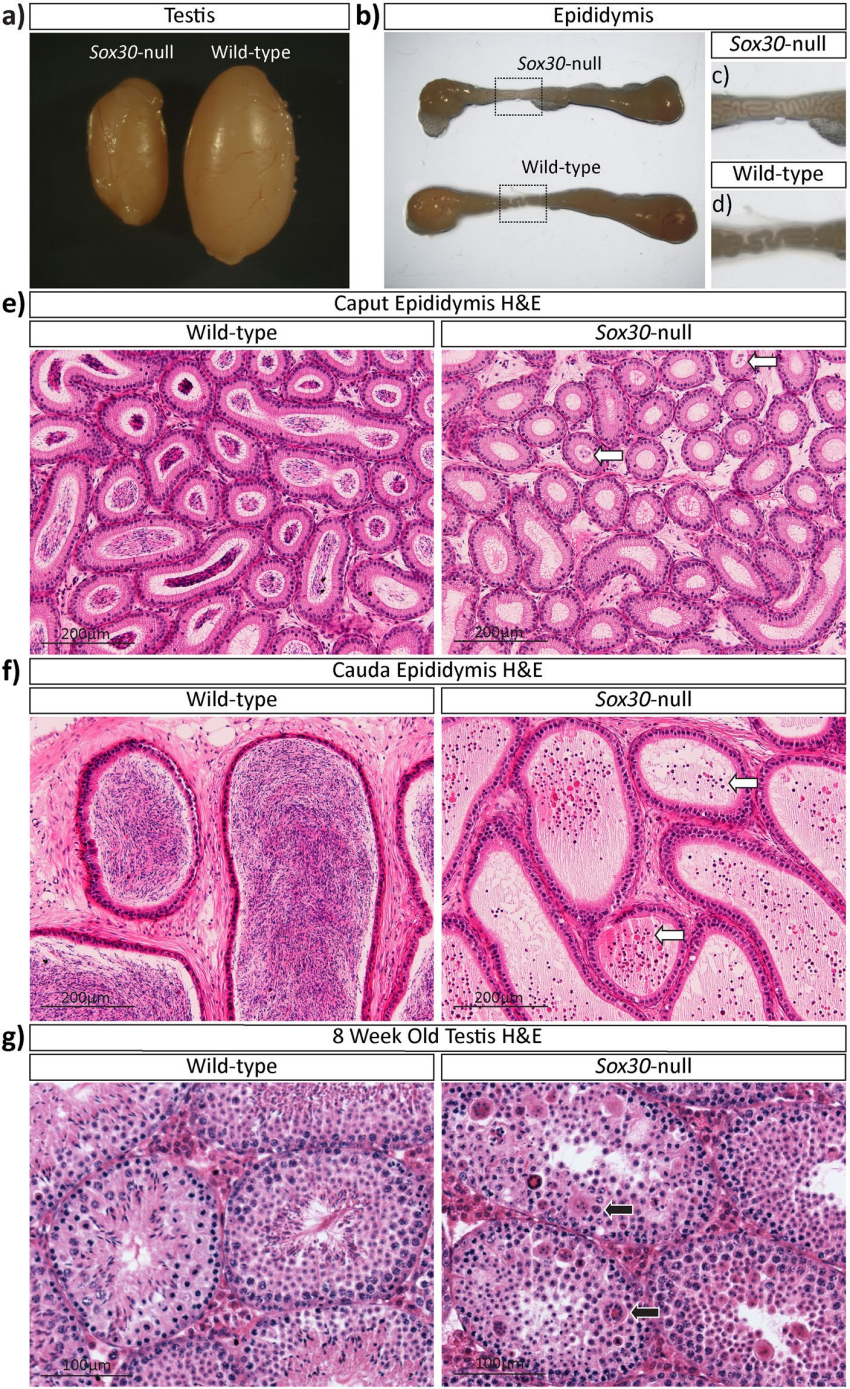
Adult *Sox30*-null testes are abnormally small and spermatids and spermatozoa are not produced. (**a**) Testes are smaller in *Sox30*-null males with (**b**,**c**,**d**) empty epididymal tubules compared to wildtype littermates (n = 3). (**e**,**f**) Sperm are found in the caput and cauda epididymides of wildtype males, but are absent from *Sox30*-null epididymides (white arrows, H&E staining). (**g**) 8-week-old testes show an absence of elongating spermatids and the presence of multinucleated giant cells (black arrows) in *Sox30*-null testis.

Following test breeding, *Sox30*-null studs were sacrificed and their testes collected for analysis. All three *Sox30*-null males had visibly smaller testes (P-value = 0.004) with an average length (5.921 mm, SD +/− 0.64, n = 3) approximately 72.4% of that of their wildtype (8.178 mm, SD +/− 0.13, n = 3) or heterozygous littermates (Fig. 2a). *Sox30*-null epididymal tubules appeared devoid of sperm when viewed using a light microscopy (Fig. 2b–d). Some abnormal germ cells (white arrows), but no sperm, were found in both the caput and cauda epididymides of *Sox30*-null males revealing that in the absence of SOX30 a significant number of haploid germ cells are precociously released from the seminiferous epithelium (Fig. 2e,f).

**Figure 3:**
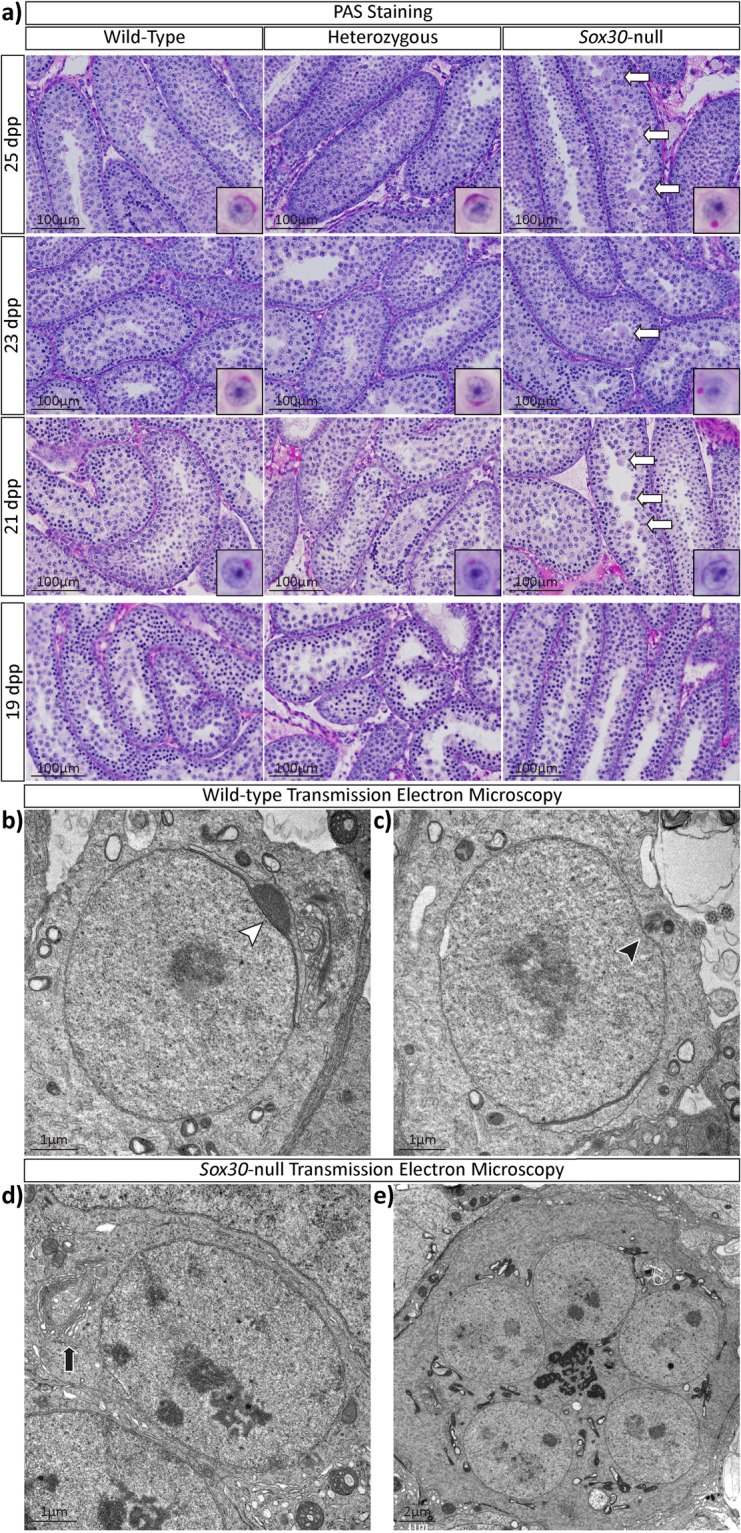
*Sox30*-null males are infertile with spermatogenesis blocked at the round spermatid stage. (**a**) PAS staining of 19, 21, 23 and 25 dpp testes reveals that abnormal germ cells (white arrows) are first seen in 21 dpp *Sox30*-null testes, a timpoint characterized by flattening of the pro-acrosome; most advanced appearance of round spermatids is shown in insert for each genotype and age. At 19 dpp no distinct granules are observed in any of the three genotypes. TEM examination of wildtype samples showing two ultrastructural features characteristic of step 3–4 spermatids: (**b**) acrosome capping of the nucleus (white arrowhead) and (**c**) docking of basal body to the nuclear membrane (black arrowhead). (**d**) These two features were not observed in *Sox30*-null round spermatids, nor were proacrosomal granules observed at the center of Golgi bodies (black arrow). (**e**) Nuclei within symplasts do not display any evidence of acrosome formation or tail development.

Sectioning and histological analysis of *Sox30*-null testes at 8 weeks of age revealed round spermatid arrest: no cells progress to elongating spermatids and later forms (Fig. 2g). Multinucleated giant cells (symplasts)^17^ were consistently present in testis cords (Fig. 2g, black arrows) and these abnormal cells were first detected at 21 dpp, an age characterized by completion of meiosis and formation of round spermatids (Fig. 3a, white arrows). The abnormal cells stain positive with periodic acid-Schiffs reagent (PAS) suggesting that proacrosomal granules have begun to form and, therefore, that they arise from early round spermatids. Such multinucleated cells, also noted in a number of other spermatogenesis mutants^18,19^, are believed to represent abnormal syncytia and form when intercellular bridges are not maintained and so widen, open and then collapse resulting in spherical structures made up of fused spermatids^20,21^. In single round spermatids of the *Sox30*-null testis, PAS staining revealed that pro-acrosomal granules form and coalesce to form a single large granule, as is normal, but that the granule never flattens to cap the nuclear surface (Fig. 3a, insets). Hence, in the absence of SOX30 round spermatids are unable to progress beyond step 3 of spermiogenesis^22^.

The above conclusions were strengthened by an analysis using transmission electron microscopy (TEM). In wildtype testes, we were readily able to observe both the docking of the basal body and the attachment of the acrosomal vesical to the nucleus, characteristic of spermatids that have progressed beyond step 3 (Fig. 3b,c). However, these features were completely absent in both the round spermatids of normal appearance and multinucleated symplasts from *Sox30*-null samples (Fig. 3d,e and Supplementary Figure 3, 4). We also noted that the Golgi bodies in *Sox30*-null spermatids were notably smaller that those in wildtype spermatids and do not appear to be producing many proacrosomal granules (Fig. 3d, black arrow and Supplementary Figure 4). As indicated in Fig. 3e (and Supplementary Figure 4) the symplasts clearly contain nuclei from multiple spermatids within a single plasma membrane. The presence of greater than four spermatid nuclei in a single plasma membrane suggests that the origin of the symplasts was not due to the failure of cytokinesis in meiosis I and meiosis II alone; wherein you would expect a maximum of 4 nuclei per plasma membrane. Rather, it suggests that symplast formation results from the coalescence of round spermatids derived from multiple spermatocytes. It is of note that, unlike somatic cells, in germ cells from spermatogonia onwards cytokinesis is incomplete and sister germ cells retain cytoplasmic bridges which are thought to be important for intracellular communication^23^. Collectively, these data show that in the absence of SOX30, spermatogenesis is unable to proceed beyond step 3 of spermiogenesis.

### Sox30 acts cell-autonomously in spermatogenesis

Although *Sox30* mRNA appears to be specific to germ cells of the fetal (Fig. 1b) and adult testis^11,12^, it is theoretically possible that deletion of *Sox30* in somatic cells, in our model, influences germ cell development. To formally exclude this possibility, we generated a *Sox30* conditional mouse line (*Sox30* ^*flox/flox*^) by crossing our *Sox30-*null line with a FLPe recombinase-expressing line^24,25^. Subsequently, we used the Vasa-Cre transgenic line^26^ to specifically delete *Sox30* in germ cells. Female *Sox30* ^*flox/flox*^ mice were mated with *Sox30* ^*flox/*+^;Vasa-Cre studs to produce *Sox30* ^Δ*/*Δ^, *Sox30* ^*flox/flox*^, *Sox30* ^*+/*Δ^ *and Sox30* ^*flox/*+^ pups. As expected, germ line specific ablation of *Sox30* in *Sox30* ^Δ*/*Δ^ pups produced the same testicular phenotype as observed in *Sox30-*null mice (Supplementary Figure 5), confirming that SOX30 acts cell autonomously in testicular germ cells.

### Some key markers of spermiogenesis are lost in the absence of *Sox30*

**Figure 4:**
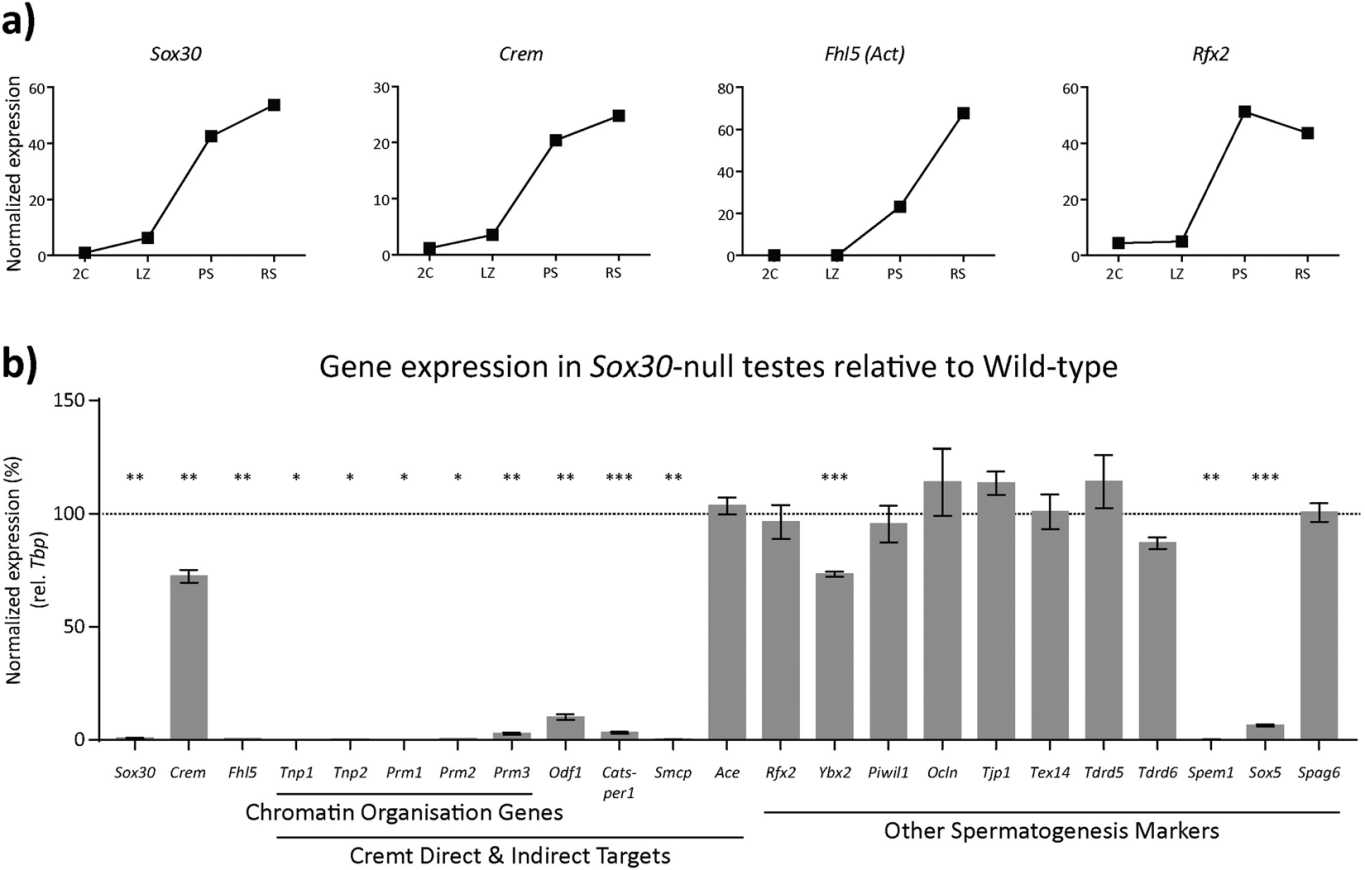
Expression of key markers of spermatogenesis in wildtype and *Sox30-null* testes. (**a**) Dynamic expression patterns of gene *Sox30*, *Crem*, *Fhl5* and *Rfx2*, which encode transcriptional activators, in purified cell populations of the mouse testis: 2 C = heterogeneous cell population containing spermatogonia and somatic cells; LZ = leptotene and zygotene spermatocytes; PS = pachytene spermatocytes; RS = round spermatids. Data was obtained by RNAseq and is publically available^27^. (**b**) qRT-PCR analysis of expression of selected spermatogenesis genes in *Sox30*-null testes collected at 25 dpp, shown relative to expression in wildtype testis. Expression is normalized to *TBP* (n = 3, two-tailed unpaired t-test; error bars represent +/− S.E.M.). *p < 0.05; **p < 0.01, ***p < 0.001.

Using recently published RNA-Seq data^27^, we compared the *Sox30* mRNA transcript profile, during the first wave of spermatogenesis, with profiles of known key spermatogenic marker genes (Fig. 4a). This analysis revealed that *Sox30* mRNA was detected in a pattern virtually indistinguishable from that of *Crem*. CREMτ (Crem-tau) isoform mRNA starts to accumulate during pachynema, is the only isoform of *Crem* expressed in the male germline at that time, and encodes a master regulator of spermiogenesis^8,28^. The *Crem*-null phenotype is similar to that of *Sox30* in that mice are healthy, females are fertile and males are sterile with spermatogenesis blocked at the post-meiotic round spermatid period. Further, as is the case for the *Sox30*-null, multinucleated cells (symplasts) are observed in the *Crem*-null testis cords. We also considered, in particular, the mRNA expression profiles of genes encoding other known transcriptional regulators. *Fhl5* and *Rfx2* mRNAs, encoding FHL5 (four and a half LIM domains protein 5, also known as ACT), a coactivator of CREM τ^29^, and RFX2, which like CREMτ is considered a master regulator of spermiogenesis^19^ were detected slightly later than *Sox30* and *Crem* (Fig. 4a). mRNA detection profiles for other key genes that underlie various features of spermiogenic progression (also extracted from published RNA-seq data)^27^ are shown (Supplementary Figure 6).

Based on these mRNA expression profiles, and information currently available about gene expression during spermiogenesis, we selected a number of marker genes and examined their expression in *Sox30*-null testes. This analysis involved qRT-PCR of wildtype and *Sox30* null testicular tissue collected at 25 dpp. This time point was chosen so as to avoid gross differences in the cell composition of tissues between genotypes: the elongation phase does not begin until step 8 and elongating spermatids are not observed in significant numbers until 28 dpp^2,30^. Extensive differences were observed in expression of spermatogenesis-related genes in the absence of SOX30 when examined at 25 dpp (Fig. 4b). Although *Crem* mRNA abundance was diminished by about 25% when analysed at 25 dpp, it was unchanged at 19 and 23 dpp (Supplementary Figure 7a,b) suggesting that *Crem* is not a direct or indirect target of SOX30. In contrast, *Fhl5* mRNA was not detected in *Sox30*-null testes at 25 dpp (Fig. 4b). This suggests that SOX30 is required for *Fhl5* mRNA expression and that any CREMτ activity that is dependent on FHL5 co-activation will be lost in the absence of *Sox30*.

We next analysed expression of genes considered to be direct, or indirect, CREMτ targets. Although deletion of *Fhl5* has no effect on expression of CREMτ targets *Tnp1* (transition protein 1), *Prm1* and *−2* (protamine 1 and −2)^31,32^, expression of these genes, and related genes *Tnp2* and *Prm3*, were essentially lost in the *Sox30*-null testis (Fig. 4b). Expression of additional CREMτ target genes *Odf1* ^33^, *Catsper1* ^34^ and *Smcp* ^35^ was lost in the *Sox30*-null testis but expression of direct CREMτ target *Ace* ^36^ was not affected (Fig. 4b). These results indicate that *Fhl5* is not the only important target of SOX30 and that SOX30 and CREMτ may share some, but not all, transcriptional targets.

Finally, we examined expression of several additional marker genes of spermatogenesis. *Rfx2*, which encodes a recently discovered transcription factor now considered a master transcriptional regulator during spermiogenesis^19^, was expressed normally in the *Sox30-*null testis while *Ybx2* (also known as Msy2)^37^, which marks specific transcripts for cytoplasmic storage, was only slightly downregulated. Similarly, loss of *Sox30* had no effect on expression of *Piwil1* (also known as Miwi)^38^ and no changes in expression were observed for *Ocln* and *Tjp1*, involved in construction and maintenance of the blood-testis barrier^39,40^ and *Tex14*, critical for the formation of intercellular bridges^41^. *Tdrd5* and *Tdrd6*, encoding Tudor domain-containing proteins involved in retrotransposon silencing and chromatoid body assembly during spermiogenesis^4,42,43^, were also expressed normally. Expression of *Spem1*, encoding a protein important for the removal of cytoplasm during spermiogenesis^44^, was lost in the *Sox30*-null testis. We examined expression of *Sox5*, another member of the SOX family of transcription factors that is relatively closely related to *Sox30* ^9^. The short 2 kb transcript of *Sox5* is detected in the post-meiotic germ cells of the adult testis as well as in the human brain^45,46^ and it has been suggested that this isoform is involved in ciliogenesis^47^. *Sox5* expression was virtually lost in the absence of SOX30, a result in keeping with our expectations based on their relative temporal expression profiles (Supplementary Figure 6). Surprisingly, *Spag6* which encodes a protein of the central axoneme of the sperm flagellum and is believed to be a direct target of SOX5^47^, was expressed normally. Overall these findings demonstrate that SOX30 is responsible, directly or indirectly, for expression of some known key participants in spermiogenesis. The full repertoire of SOX30-dependent genes, and the overlap of this set with genes downstream of CREMτ, remains to be determined.

## Discussion

Our findings demonstrate that the SOX family member SOX30 has an indispensable role in spermatogenesis, in mice. Homozygous deletion of *Sox30* results in male sterility with spermatogenesis completely blocked in early round spermatids. Staining of testis sections with PAS and further investigation with TEM showed that the round spermatids fail to process beyond step 3 of spermiogenesis, a transition characterised by the attachment and flattening of the pro-acrosomal granule around the nucleus and the initiation of spermatid tail growth and attachment^22^. In addition to the spermatogenic block, a large number of multinucleated giant cells, also known as symplasts, were present: these are likely to be of early round spermatid origin as the cells contain multiple scattered granules of various sizes that stained positively for PAS.

Despite our initial hypothesis, that SOX30 is involved in meiosis, we did not find any evidence that this is so, in either sex. In the fetal ovary, *Sox30* expression is initiated very shortly after *Stra8* yet, in the *Sox30*-null model, we found no evidence that meiosis is perturbed. In the male, meiosis appears to proceed unimpeded as spermatocytes are able to develop into post-meiotic early round spermatids. It is not until later, at the round spermatid phase, that abnormalities are first observed. The production of *Sox30* mRNA transcripts well before the protein is required is in agreement with observations for spermatogenesis-associated genes in general: it was recently concluded that genes initiating at pachynema tend to function in post-meiotic rather than meiotic processes^27^. This is in line with evidence that many mRNAs are produced, but then subjected to translational delays, until the relevant protein is required later in spermiogenesis^48,49^.

The exact role that SOX30 plays in spermiogenesis is not yet clear. What we know thus far is that, in the absence of SOX30, *Crem* mRNA is still expressed relatively normally. The *Crem*τ isoform encodes a master transcriptional regulator that induces transcription of numerous post-meiotic genes in round spermatids^50,51^ and we reveal here that its temporal expression profile during spermatogenesis is extremely similar to that of *Sox30*. Homozygous *Crem* mutants have a phenotype strikingly similar to that of *Sox30* null mutants, with a complete spermiogenic block at round spermatids and the formation of symplasts^18,52^. The expression of several genes considered direct or indirect targets of CREMτ is essentially lost in the *Sox30*-null testis (*Tnp1* and *−2*, *Prm1*, *−2* and *−3*, *Odf1*, *Catsper1*) whilst at least one other is expressed normally (*Ace*). Thus, it seems likely that SOX30 works either cooperatively or in parallel with CREMτ to drive transcription that is necessary for round spermatid maturation. Because these changes are observed at 25 dpp, before elongating spermatids normally emerge, we can conclude that the transcriptional changes observed reflect the loss of direct or indirect targets of SOX30 rather than absence of a particular cell type. Similar trends were also observed at 23 dpp but with lower robustness due to the normally low expression of these genes at this time point (Supplementary Figure 7b)

Publicly available data suggest that SOX30 is unlikely to be a direct or indirect target of CREMτ: no change in *Sox30* expression was detected in the *Crem-*null testis^32,53^. Although a half Cre site was identified 87 bp upstream of the *Sox30* transcription start site in ChIP-seq studies, this was one of more than 6700 genomic loci found to be bound by CREMτ in mouse male haploid germ cells^54^. It is thought that round spermatids have a particularly accessible chromatin environment which possibly explains the large number of CREMτ-bound loci, many of which correspond to genes that are never expressed during spermiogenesis^54^. Given that CREMτ clearly occupies many more promoters that it actually regulates it seems plausible that co-regulation by SOX30, which is expressed in a highly cell type-restricted manner^11,12^ this study, lends specificity to transactivation instigated by CREMτ. The details of any physical or functional interactions between CREMτ and SOX30 remain to be determined.

Expression of *Flh5*, which encodes a coactivator for CREMτ, is lost in the absence of *Sox30*. Despite strong evidence for the coactivator function of FLH5^29^ the spermatogenic phenotype of the *Fhl5*-null (fewer mature germ cells and abnormal tail and head morphology) is considerably less severe than that of the *Crem-*null (complete block in spermiogenesis)^55^. Presumably this means that FLH5 functions as coactivator for only a subset of CREMτ post-meiotic target genes^31,55^. Given the *Fhl5* null phenotype, loss of *Fhl5* expression in the *Sox30*-null testis is not sufficient, by itself, to account for the severe spermatogenic block we observe.

RFX2 is considered a second master transcriptional regulator of spermiogenic gene expression^19^. *Rfx2*-null mice have a phenotype resembling that of the *Sox30*-null, including defective spreading/adhesion of the acrosomal cap and the formation of giant multinucleated cells. RFX2 seems to be particularly important for the transcription of genes involved in cilia assembly and function and the set of RFX2 target genes is distinct from those regulated by CREMτ^19^. Although the onset of *Rfx2* expression looks to be slightly later than that for *Sox30* (Fig. 4a), *Rfx2* expression is not affected by *Sox30* deletion and, reciprocally, our examination of publicly-available data shows that the expression of *Sox30* is unaffected in the *Rfx2*-null^19^. Hence it seems that *Rfx2* and *Sox30* are independently regulated.

Other than *Crem*-null and *Rfx2*-null models, described above, mouse mutants showing complete blockage of spermatogenesis at the round spermatid phase are rare^56^. One additional genetic mutant that arrests in early spermiogenesis is the homozygous deletion of *Piwil1* (*piwi-like RNA-mediated gene silencing 1*, also known as *Miwi*)^38^. In the *Piwil1*-null round spermatids are blocked at a similar developmental period to those in the *Sox30*-null testis, with only a small number of spermatids proceeding to step 4 of spermiogenesis. Spermatids with pyknotic and fragmented nuclei were observed in *Piwil1* mutant gonads, however, the presence of multinucleated cells was not mentioned. As is the case for the *Sox30* mutant, *Piwil1*-null females are fertile. The cytoplasmic RNA binding protein PIWIL1 binds to *Fhl5* mRNA as well as to mRNAs of target genes of CREMτ although, in *Piwil1*-null mice, expression of *Crem* itself is not affected. We found that *Piwil1* expression was normal in the *Sox30*-null testis.

Using GeneChip RNA profiling data and mass spectrometry, others have shown human SOX30 expression is highly specific to the adult testis^12^. Expression of *SOX30* was not detected above baseline levels in any adult tissue except the testis whilst SOX30 protein was found at high levels only in adult testis in data obtained by mass spectrometry. Those authors hypothesized that SOX30 is important in spermatogenesis and that it might act as a potential hub protein as it is predicted to have at least 13 interacting proteins. Given this apparent specificity of expression, and our findings in mice - that deletion of *Sox30* results in healthy individuals with fertile females and sterile males - we speculate that mutations in *SOX30* could easily be retained in the population and, therefore, underlie some cases of male infertility. It is interesting to note that despite the apparent restriction of SOX30 expression to the germ cell lineage it has been suggested that SOX30 is ectopically expressed in lung cancer and acts as a tumour suppressor, by virtue of its ability to directly activate transcription of *Trp53*, a major cell-cycle regulator^57^.

Approximately 10–15% of couples are infertile and a male factor is involved in almost half of these cases. Male infertility encompasses a wide variety of disorders but the most severe presentation is nonobstructive azoospermia (NOA), a condition where spermatogenic failure has occurred and spermatozoa are completely lacking^58^. NOA is found in about 5 to 10% of male infertility patients and cannot be overcome by use of approaches such as *in vitro* fertilization and intracytoplasmic sperm injection. Thus, there is a compelling need to gain a better understanding of the molecular mechanisms that underlie normal mammalian sperm production: such knowledge will inform the development of new diagnostics, targeted therapies and the production of gametes *in vitro* as well as efforts to develop male-based nonhormonal contraceptive methods for humans and animals. Given our findings, that we speculate that *SOX30* mutations may underlie some of the cases of idiopathic round spermatid arrest in humans.

## Methods

### Animal Ethics

All procedures involving animals and their care were carried out in accordance with institutional, state and national guidelines. This study was approved by the University of Queensland and Monash University Animal Ethics Committees.

### Mice

For analysis of fetal and postnatal expression of *Sox30*, gonadal tissue samples were obtained from an X-linked-eGFP mouse line^59^ maintained on a Swiss Quackenbush background. An Oct4ΔPE:eGFP mouse line (OG2)^14^ on a C56BL/6 J background was used to segregate germ and somatic cells via fluorescence activated cell sorting (FACS) as previously described^60^. Sox30tm1a(KOMP)Wtsi targeted embryonic stem (ES) cells were obtained from Knockout Mouse Project (KOMP) ^25^repository (UC Davis) and *Sox30*-null mice were produced, using these cells, by the Australian Phenomics Network (APN, Monash node)^61^. The modified *Sox30* allele carried by the *Sox30* knockout mice consists of a gene trap cassette inserted into the first intron of *Sox30*. This cassette includes a mouse *En2* splice acceptor followed by *lacZ* reporter transgene with a SV40 polyadenylation sequence: this creates a null allele. This initial null allele also contains *FRT* sites which are positioned so as to allow excise of the gene trap cassette upon exposure to FLP recombinase, leaving behind *loxP* sites flanking the second and third exon of *Sox30* (thus generating a *Sox30* conditionally null allele). Thus, we generated a conditional knockout (*Sox30* ^*flox/flox*^) mouse line by crossing *Sox30*-null mice with FLPeR mice^24^. For experimental analysis *Sox30* ^*flox/flox*^ mice were bred with VASA-Cre transgenic mice, which express *Cre* recombinase in germ cells from approximately 15.5 dpc onwards^26^. Genotyping of mice was performed by PCR using the primers indicated in Supplementary Table S1 online with the exception of the X-linked-eGFP mice which were genotyped by virtue of their green fluorescence under UV light.

### Timed matings and tissue collection

X-linked-eGFP and homozygous Oct4ΔPE:eGFP studs were housed with wildtype females (Swiss Quackenbush and C57BL/6 J respectively) for timed matings with noon of the day when the copulatory plug was observed designated as 0.5 dpc. To investigate the *Sox30*-null phenotype, males and females heterozygous for the *Sox30-*null allele were mated. For germ cell-specific ablation of *Sox30*, *Sox30* ^*flox/flox*^ females were mated with *Sox30* ^*flox/+*^;VASA-Cre^+^ males. The sex of the embryos was identified by visual inspection of dissected gonads or, in the case of X-linked-eGFP, by presence or absence of fluorescence of the whole embryo. *Ube1* genotyping using tail tissue^62^ was retrospectively preformed to reconfirm the sex of all collected samples. For tissues to be analysed by qRT-PCR, gonad pairs were dissected free of the adjacent mesonephric tissue and the gonads were placed immediately in RNAlater (QIAGEN, #76106) for storage. When collected for qRT-PCR postnatal testes were first decapsulated before storage in RNAlater. For embryonic tissues to be analysed by histology, whole embryos (minus tail used for genotyping) were collected, fixed in 4% paraformaldehyde in phosphate buffered saline (PFA/PBS), dehydrated and embedded in paraffin for sectioning. When collected for histological analysis the capsule of postnatal testes was pierced in multiple locations with a 30 G needle and the samples were fixed with Bouin’s solution (Sigma, #HT10132) or 4% PFA/PBS before being processed and embedded in paraffin for sectioning.

### Quantitative reverse transcriptase PCR (qRT-PCR)

Total RNA was extracted from individual gonads or gonad pairs using RNeasy Micro Kit (Qiagen, #74004) or RNeasy Mini Kit (Qiagen, #74106) including on-column DNase treatment. Total RNA-containing eluate was immediately used for cDNA synthesis by reverse transcription using a High Capacity cDNA Reverse Transcription Kit (Applied Biosystems, #4368813). For fetal gonadal samples and the time course of *Sox30* and *Stra8* expression in the postnatal testis qRT-PCR was performed on a Viia7^TM^ Real-Time PCR System (Applied Biosystems). Expression levels of *Dmc1* (Mm00494485_m1), *Rec8* (Mm00490939_m1), *Sox30* (Mm00557681_m1), *Stra8* (Mm00486473_m1) and *Sycp3* (Mm00488519_m1) relative to *Ddx4* (Mm00802445t_m1) or *Tbp* (Mm01277045_m1) were quantified using Taqman Gene Expression Assays and Universal Taqman Master Mix (Applied Biosystems, #4318157). To investigate the effects of *Sox30* ablation in 19 dpp and 25 dpp testis, qRT-PCR was performed on a Quantstudio6 and Quantstudio7 (Applied Biosystems) respectively using primers listed in Supplementary Table S2 online and SYBR Green PCR Master Mix (Applied Biosystems, #4309155). Relative cDNA levels were determined by the 2^−ΔCT^ method. All kits and assays were performed according to instructions supplied by the manufacturer.

### Histological sectioning and staining

Whole embryos and postnatal testes embedded in paraffin were sectioned at 7 µm and dewaxed by immersion into xylene twice for 10 minutes and then rehydrated through an ethanol series ranging from 100% to 35% ethanol (v/v) in water. Histological staining of Bouin’s fixed samples was performed with either hematoxylin and eosin (H&E, Sigma, #HHS32 and #HT110332, respectively) or periodic acid-Schiff reagents (PAS, Sigma, #395B-1KT). All staining procedures were carried out as per manufacturers’ instructions. In our attempts to visualize SOX30 protein by immunofluorescence we used SOX30 antibodies ab26024 and ab71033 (Abcam), sc-20104 and sc-390333 (Santa Cruz Biotechnology) and 13017-AP (Proteintech) without success.

### *In-situ* hybridisation

The 3′ UTR of *Sox30* was PCR amplified from postnatal testis cDNA using primers 5′-ccctttggctatggaaattttcc-3′ and 5′-caatgcataccaaatgggaaaga-3′ and cloned into pGEM-T-easy (Promega). Resulting clones were verified by Sanger sequencing and then used for DIG labelled RNA probe synthesis as previously described^63^. 4% PFA fixed postnatal testes embedded in paraffin were used for *in-situ* hybridisation using previously described protocol^64^ with adaptations to utilise and detect DIG-labelled probe. Briefly, DIG-labelled probes were hybridised at 1μg/ml and detected by blocking the slides with 10% heat-inactivated sheep serum (HISS) in NT buffer (50 mM Tris-HCl and 150 mM NaCl at pH 7.5) for at least 1 hour before incubating overnight with 1:2000 dilution of α-DIG-AP Fab fragments (Roche) in 1% HISS/NT at 4 °C. The Fab fragments were rinsed off with 3 washes using NT buffer and the slides were then placed in NTM buffer (100 mM Tris-HCl, 100 mM NaCl and 50 mM MgCl_2_ at pH 9.5) for 10 minutes to equilibrate. A staining solution consisting of 175μg/ml BCIP (Roche) and 350μg/ml NBT (Roche) in NTM was applied to the slides and colour development was check at regular intervals by placing the slides in NTM. Once the desired staining intensity has been reached the slides were fixed with 4% PFA and mounted with an aqueous mounting media.

### Transmission electron microscopy

Freshly dissected testis were fixed in a solution of 100 mM Sodium Cacodylate buffer with 4% PFA, 5% glutaraldehyde and 0.02% picric acid for at least 3 hours then bisected for fixation overnight at 4 °C. After fixation the samples were washed three time with 100 mM Sodium Cacodylate buffer before preforming a secondary fixation with 2% Osmium tetroxide in 100 mM Cacodylate buffer for 2 hours. The samples were rinsed with 3 washes of MilliQ water before staining with 1% Uranyl Acetate for 90 minutes and then rinsed with another 3 washes of MilliQ water. Samples were dehydrated in an acetone series of 50%, 70%, 90% and three 100% acetone washes. Araldyte Epon resin was gradually introduced, first with a wash of 50:50 resin:acetone mix for 2 hours then a 70:30 resin:acetone mix overnight on rollers. The samples were soaked in three changes of 100% Araldyte Epon for 2 hours before being embedded and polymerisation at 60 °C for 48 hours. Samples were sectioned at 90 nm, mounted onto carbon coated copper grids and double stained with uranyl acetate and lead citrate.

### Data availability

The datasets generated during and/or analysed during the current study are available from the corresponding author on reasonable request.

## Electronic supplementary material

Dataset 1
