# Supplementary material for: SOX30 is required for male fertility in mice
Source: Sci Rep. 2017 Dec 15;7:17619. doi: 10.1038/s41598-017-17854-5 (PMC5732304; doi:10.1038/s41598-017-17854-5)
Supplement: Supplementary file 1 — Dataset 1 [file 41598_2017_17854_MOESM1_ESM.doc]

# Supplementary Material

# SOX30 is required for male fertility in mice

## Authors and affiliations

Chun-Wei Allen Feng^1,2^, Cassy Spiller^1^, Donna J. Merriner^3^, Moira K. O’Bryan^3^, Josephine Bowles^1,2*#^ and Peter Koopman^2#^

1. School of Biomedical Sciences, The University of Queensland, Queensland, Australia

2. Institute for Molecular Bioscience, The University of Queensland, Queensland, Australia

3. School of Biological Sciences, Monash University, Victoria, Australia

# equal contributors, listed alphabetically

* corresponding author

# Supplementary Figures


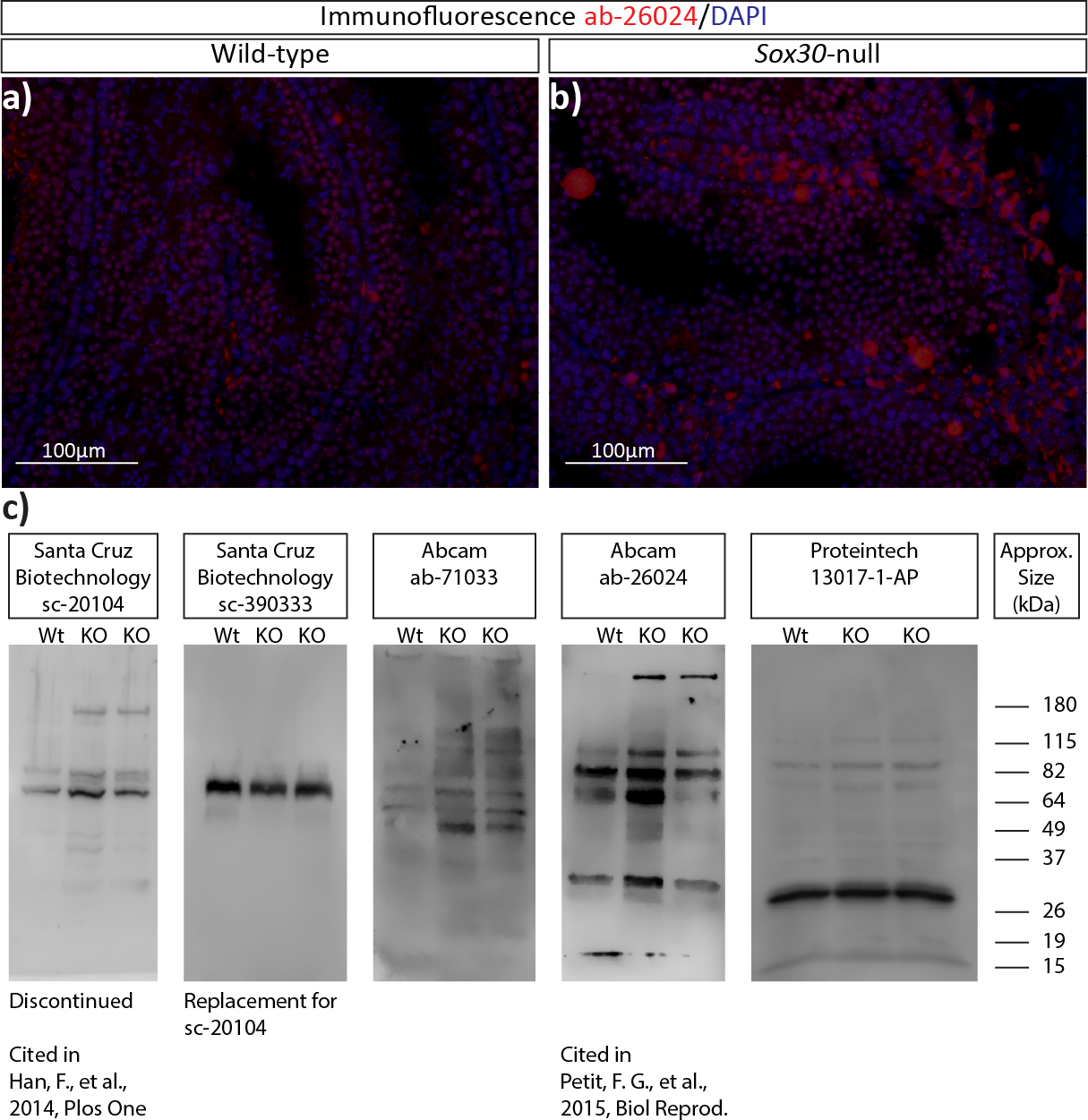


Supplementary Figure 1: Commercially available SOX30 antibodies lack specificity.

We stained for SOX30 by immunofluorescence using SOX30 antibodies ab26024 (Abcam), ab71033 (Abcam) and sc-20104 (H-300, Santa Cruz Biotechnology). In our hands, we were unable to observe any staining using the two latter antibodies in foetal ovaries nor post-natal testis, but with ab26024 we observed spermatid-specific staining in both a) wildtype and b) *Sox30*-null testis, suggesting that this staining was not specific to SOX30. c) The three antibodies, along with two others (sc-390333 (Santa Cruz Biotechnology) and 13017-1-AP (ProteinTech)), also gave non-specific results when used in Western blots on wildtype (Wt) and *Sox30*-null (KO) testis extracts (predicted M.W. 82-84kDa).


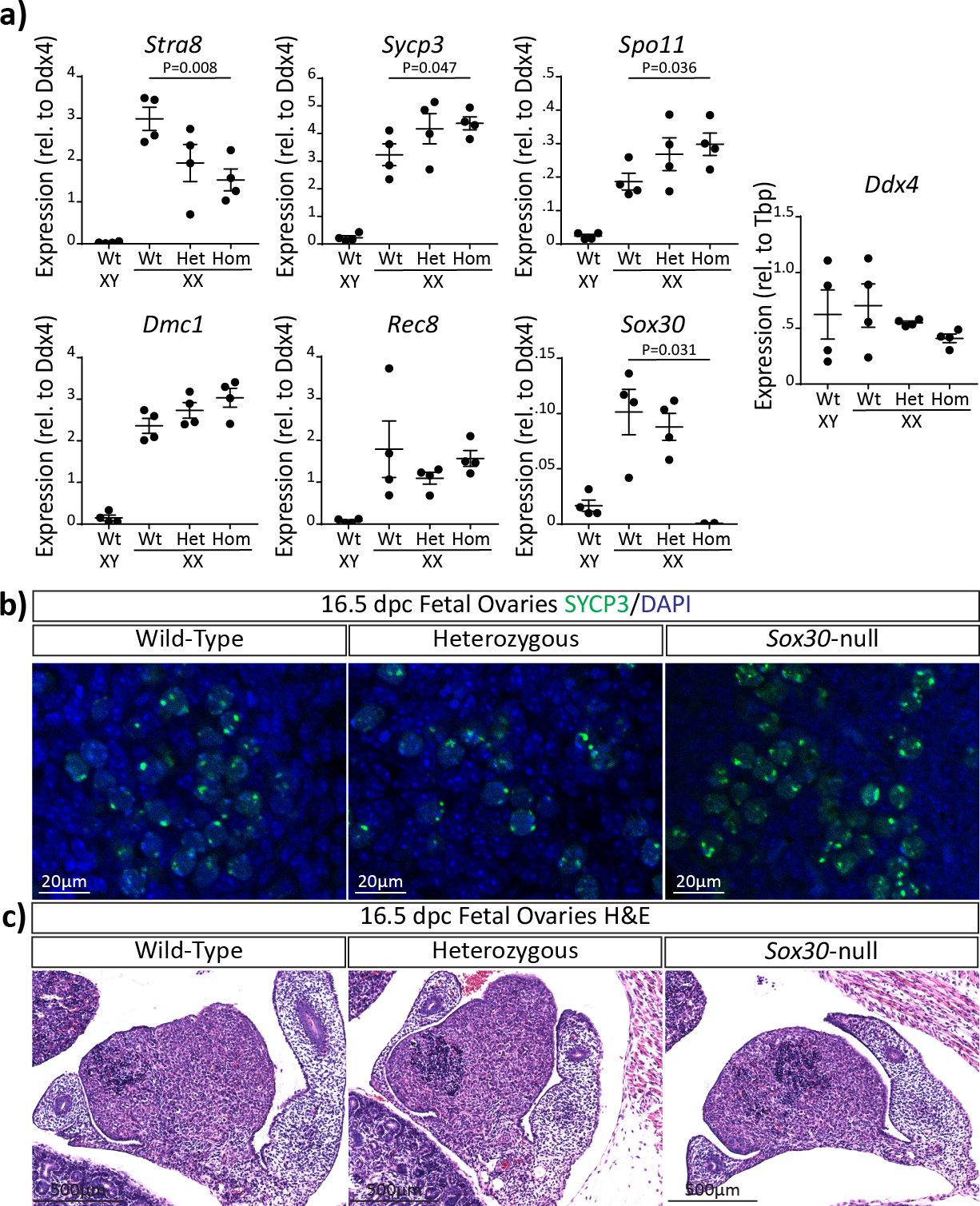
Supplementary Figure 2: Meiosis proceeds normally with minor changes to some meiotic gene expression in foetal ovaries of the *Sox30*-null mutant.

a) Expression of *Stra8* is lower in ovaries of *Sox30*-null mutants (Hom) at 15.5 dpc but expression of other meiotic markers, *Sycp3, Spo11, Dmc1* and *Rec8* is not diminished and, for *Sycp3* and *Spo11*, is slightly increased. The expression of *Sox30* is diminished in the heterozygous (Het) mutants and is absent from homozygous mutants as expected (two-tailed unpaired t-test; n=4; error bars represent S.E.M.). b) Immunofluorescence imaging for SYCP3 shows that the formation of synaptonemal complex is normal in *Sox30*-null ovaries at 16.5 dpc. c) The general ovarian morphology of histological sections stained with H&E is normal.


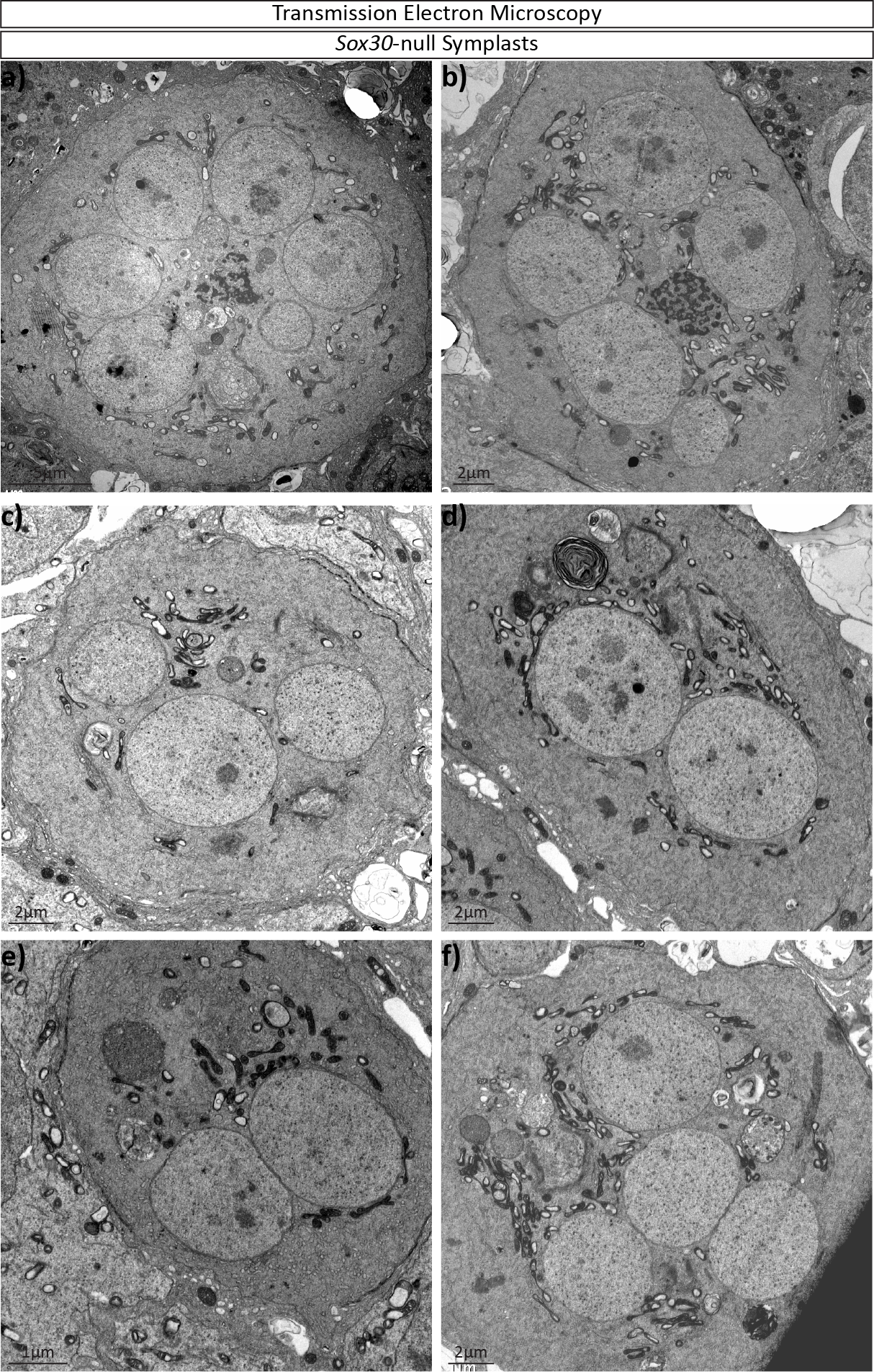


Supplementary Figure 3: Multinucleated symplasts found in adult *Sox30*-null testis lack signs of acrosome and tail development.

a-f) Representative examples of multinucleated symplasts found in *Sox30*-null adult testes showing a variety of abnormal ultrastructures. Importantly, none of the symplasts examined possessed two key features of early spermatid development: the attachment of the acrosomal granule to the nucleus and docking of the basal body.


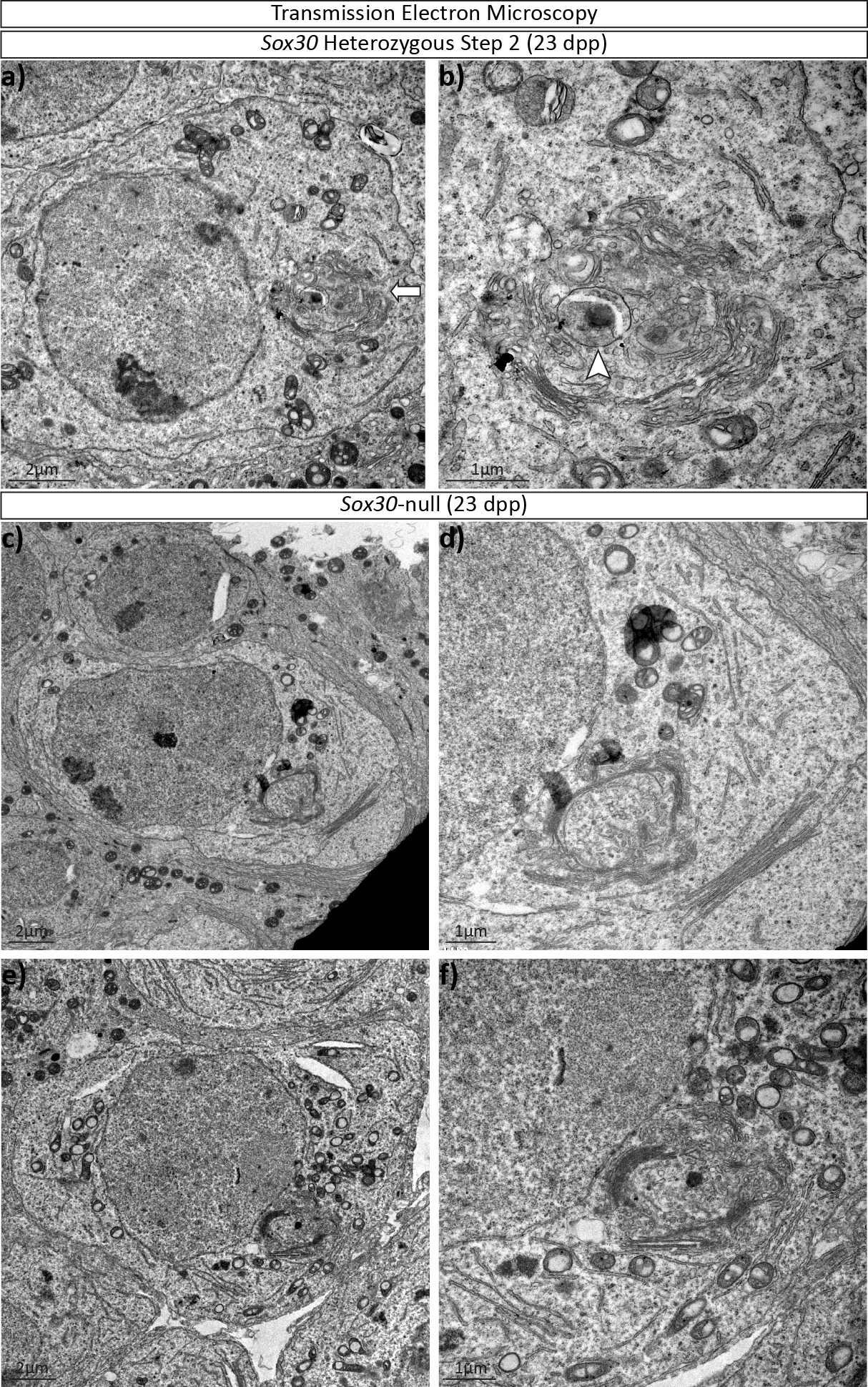


Supplementary Figure 4: Golgi bodies of *Sox30*-null round spermatids lack features of acrosomal vesicle formation.

a) An example of a normal step2-3 spermatid found in a *Sox30* heterozygous sample at 23 dpp showing the formation of an acrosomal vesicle adjacent to the nucleus (arrow); b) Magnified view of the forming vesicle showing the acrosomal granule at the centre of the Golgi apparatus (arrowhead). No proacrosomal granules were observed at the centre of the Golgi body in the *Sox30*-null sample shown in Figure 3d. c) & e) Two more examples are provided here from 23 dpp *Sox30*-null samples with magnified views d) & f) focusing on the Golgi bodies.


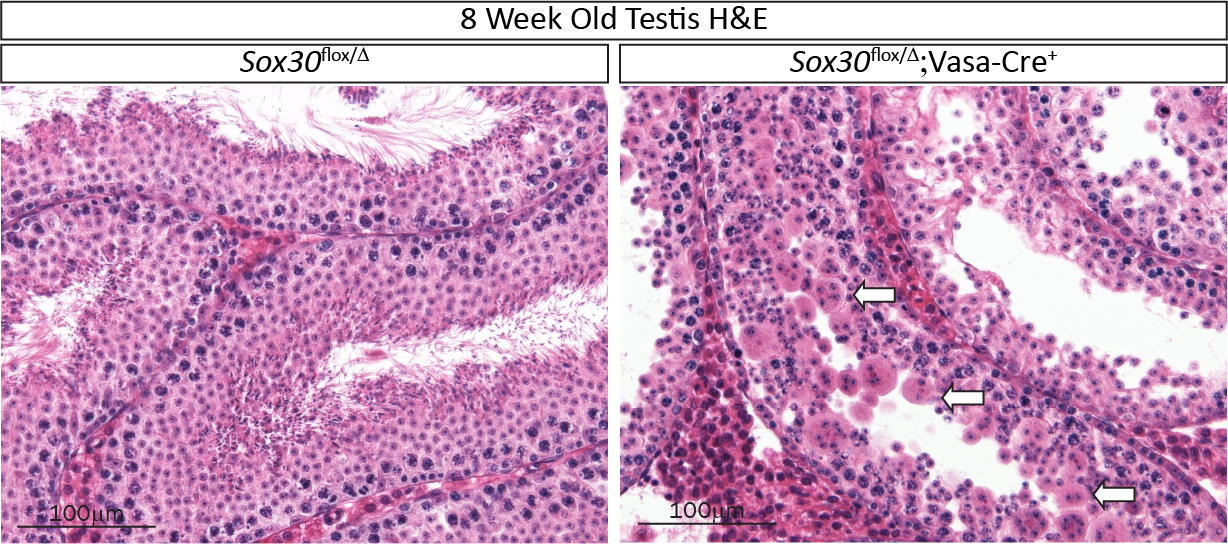


Supplementary Figure 5: The effect of *Sox30* deletion is cell autonomous, as demonstrated by germ cell-specific ablation of *Sox30*

H&E staining of 8-week-old *Sox30* Δ/flox and Vasa-Cre transgene positive testes (right) reveals spermatogenic arrest at the round spermatid stage. As is the case for the ubiquitous *Sox30*-null (Figure 2g), multinucleated giant cells are observed (white arrows). Control testis tissue (*Sox30* flox/Δ without Vasa-Cre transgene, left) appears histologically normal.


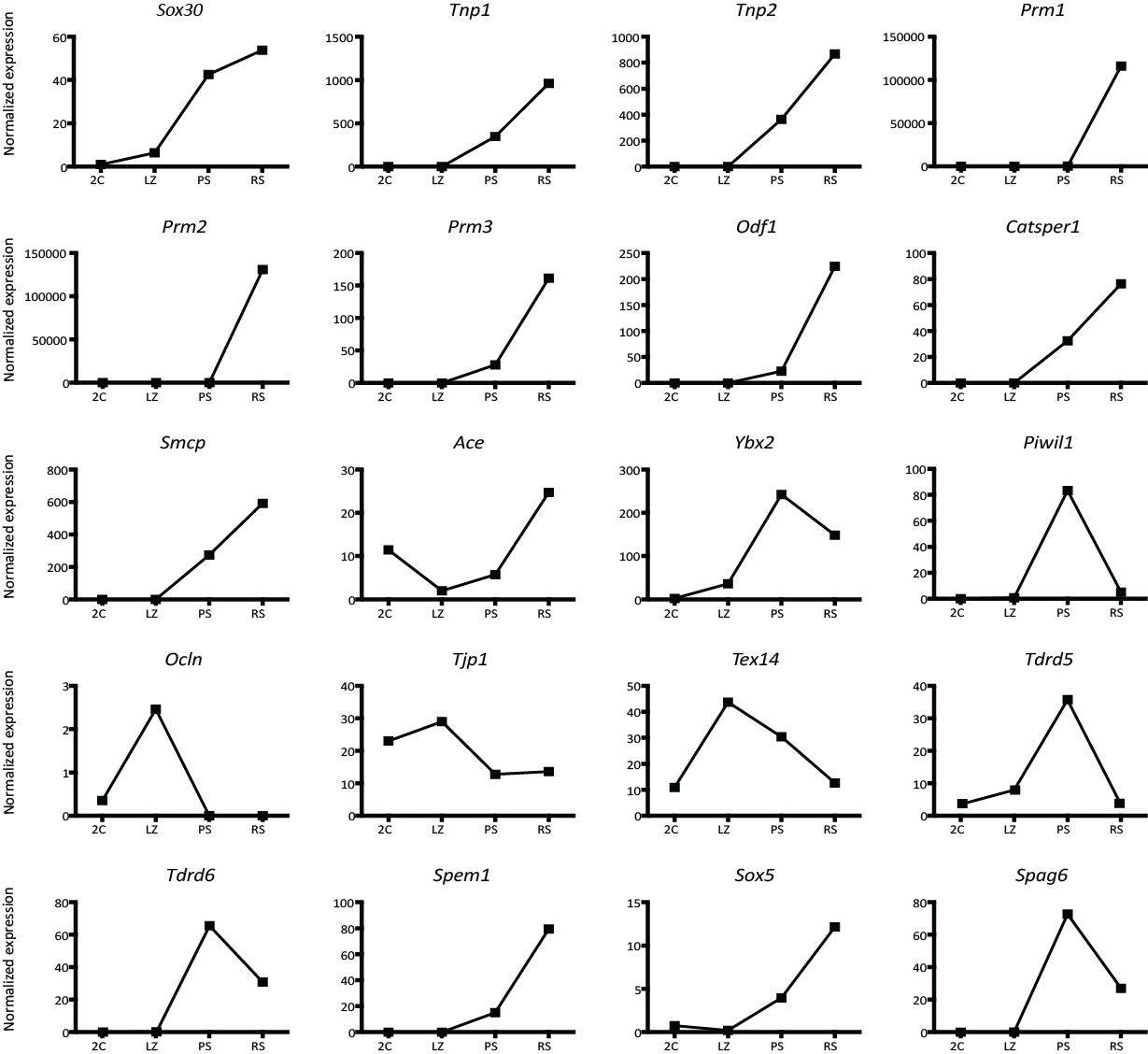


Supplementary Figure 6: Dynamic expression patterns of selected spermatogenic markers in purified cell populations.

Expression data, obtained by RNA-Seq of purified cell populations of the mouse testis, was extracted from publicly-available information {da Cruz, 2016 #423}. 2C = heterogeneous cell population containing spermatogonia and somatic cells; LZ = leptotene and zygotene spermatocytes; PS = pachytene spermatocytes; RS = round spermatids. In addition to profiles for *Sox30* and key transcriptional activators (as also shown in Fig. 4a), profiles for genes chosen for expression analysis by qRT-PCR (Fig. 4b) are presented.


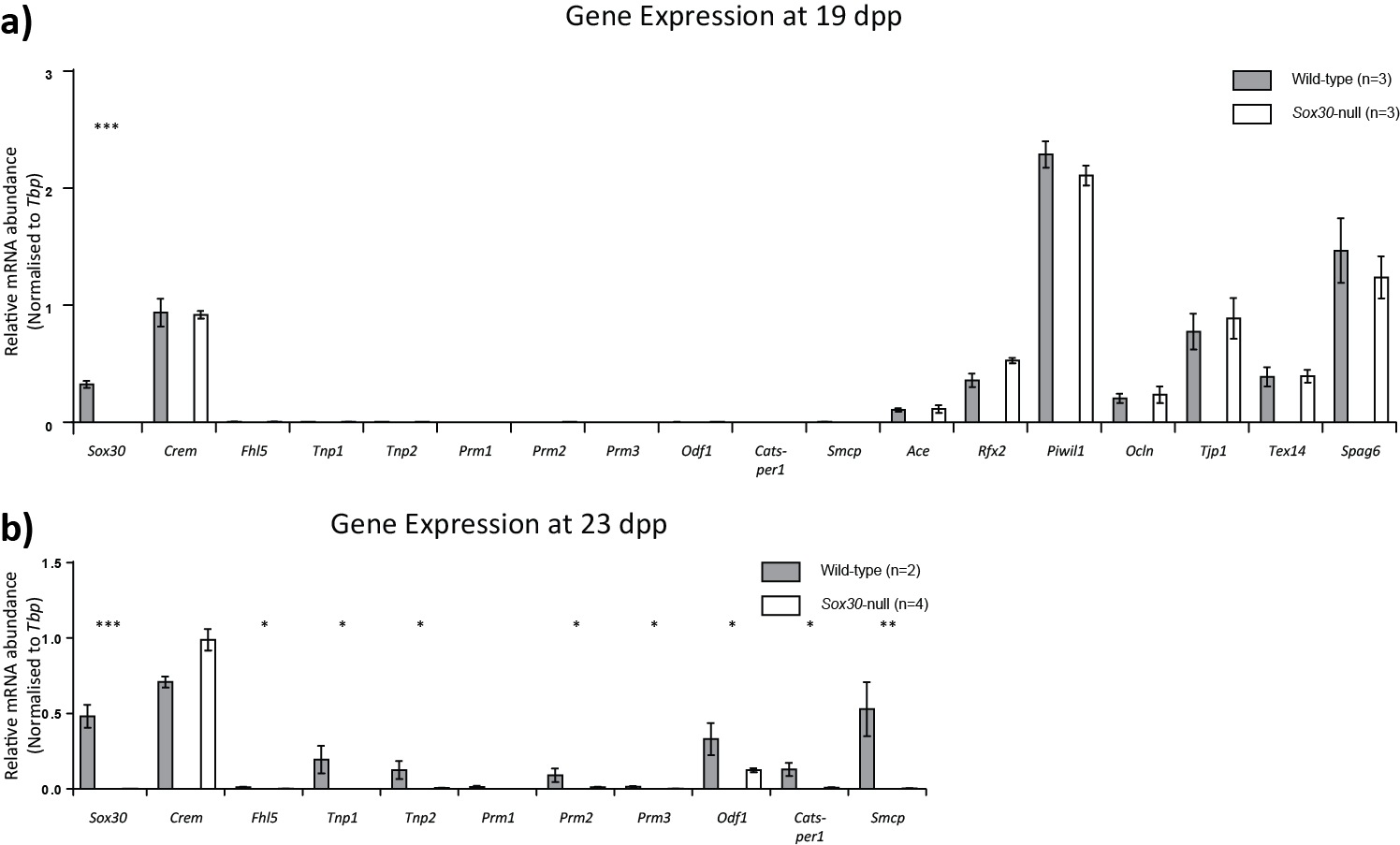


Supplementary Figure 7: Expression of key markers of spermatogenesis in wildtype and *Sox30*-null testis at 19 and 23 dpp.

a) qRT-PCR analysis of expression of selected spermatogenesis genes in wildtype and *Sox30*-null testes collected at 19 dpp and b) 23 dpp. Expression is normalized to TBP (n values as specified in graph legend, two-tailed unpaired t-test; error bars represent S.E.M.). *p<0.05; ** p<0.01, *** p<0.001.

Supplementary Table S1: Genotyping Primers

| **Name** | **Sequence (5’ to 3’)** | **Application** |
| --- | --- | --- |
| Oct4dPE_Fv2 | CCAGTAATGGGATCGTGA | Oct4ΔPE:eGFP transgene |
| eGFP_Rv1 | CGTCGCCGTCCAGCTCGACCAG |  |
| Sox30_WTF | CATATGTATAATTTTCCTCCCAGG | Sox30 wildtype and conditionally floxed |
| Sox30_WTR | GATTCCAAAATCCCTCATAGTC | alleles |
| Sox30_KOR | CAACGGGTTCTTCTGTTAGTCC | Used with Sox30_WTF for KO-first allele |
| Sox30flox_R | GCGAGCTCAGACCATAACTTCG | Used with Sox30_WTF for deleted allele |
| FLP_F | CAGTTCGAATCATCGGAAGAAGCAG | FLPeR transgene |
| FLP_R | GTCATCAAATGTCTTCCAATGTGAG |  |
| Cre_5F | CTGACCGTACACCAAAATTTGCCTG | VASA-Cre transgene |
| Cre_6R | GATAATCGCGAACATCTTCAGGTTC |  |
| Ube1_F | TGGTCTGGACCCAAACGCTGTCCACA | Sexing of embryos and neonates |
| Ube1_R | GGCAGCAGCCATCACATAATCCAGATG |  |

Supplementary Table S2: qRT-PCR Primers for Spermatogenesis

| **Gene Name** | **Forward Primer (5’ to 3’)** | **Reverse Primer (5’ to 3’)** |
| --- | --- | --- |
| *Tbp* | ACGGACAACTGCGTTGATTTT | ACTTAGCTGGGAAGCCCAAC |
| *Sox30* | CCCTGACACCAGTGCCTATT | TCCTTACTGAAGGGAGTATCAGGT |
| *Crem* | CTTTCACGAAGACCCTCATATAGAA | TGGTAGCAATGTTAGGTGGTGT |
| *Fhl5* | AAAGCCTTTCGTTGCCAAG | AAATTCCATTTTCCGAGAACC |
| *Rfx2* | GTGGGGACGCTGTCTACG | CCTGGAGTCTCAAAGTAAGATGC |
| *Tnp1* | CATCACAAGTGGGATCGGTA | TCAAGAGAGGTGGAAGCAAGA |
| *Tnp2* | GAGCCTTCCCACCACTCAT | TGCACTGGTTACTGGTGTGACT |
| *Prm1* | ATGGCCAGATACCGATGCTG | GCAGCATCTTCGCCTCCTC |
| *Prm2* | ACAAGAGGCGTCGGTCAT | CTCCTTCGGGATCTTCTGC |
| *Prm3* | AGACAGGAGTGGGCGATG | GCTTCTTCATGGAGGACTCG |
| *Odf1* | TGTGGCCTGTGTGACCTCTA | TTCTATTTGTCGTCCTTCTGAGTCT |
| *Catsper1* | CTGAGCTAGAGATCCGAGGTG | CAATTAGCTTGAGGACTGCTTCT |
| *Scmp* | AGAGCCCAAGGAAGAACTGTG | GTGGACAGCACGGTTTAGGT |
| *Ace* | TCTGCTTCCCCAACAAGACT | AGGATGTTGGTGAGCTCTGG |
| *Ybx2* | TTCTGCGGAGTGTTGGAGAT | AGGCCCAGTGACATTAGCAG |
| *Piwil1* | CCTGCGGCAGTTGTAGGA | CTCGGCCAGTCATTTTCC |
| *Ocln* | GTCCGTGAGGCCTTTTGA | GGTGCATAATGATTGGGTTTG |
| *Tjp1* | TTTGAGAGCAAGCCTTCTGC | AGCATCAGTTTCGGGTTTTC |
| *Tex14* | CAGGAGCTACTTGATGAAATTGAG | CGTGCCGTGTTCTCATGT |
| *Tdrd5* | ACAAGAAGCCTAATCTGGTGGT | TGCGGTTTGTTTGTTTCTACC |
| *Tdrd6* | CGAACTAGCCGAAGAAGGAAC | CAGACATTCTCGGGGCTTAC |
| *Spem1* | GTATTAACATTGGCATCAATTTGG | GGTGTAAGAGGATGCGGATT |
| *Sox5* | TGCGTATCGGGGAGTACAA | GATGGGGATCTGTGCTTGTT |
| *Spag6* | CAGGAATACATCAACAGCATCAA | TGTATCGGAGTATCCAGGTGAA |

# Supplementary Methods

## Western Blotting

Whole testes were disassociated in 1ml RIPA buffer supplemented with 1x cOmplete protease inhibitor cocktail (Roche). Prior to loading, the samples were denatured for 3 minutes at 100°C and the placed immediately on ice. 30μl plus 10μl 4x loading buffer was loaded into each well of a 10% acrylamide gel with 4% stacking gel on top and ran at 100V for approximately 1 hour. The gel was assembled into a blot transfer sandwich with PVDF membrane and wet transferred at 50V for 1 hour. The PVDF membrane was washed three times with TBST (50mM Tris-HCl, 0.1% Tween-20, pH7.6) and blocked for 1 hour with 5% skim milk powder in TBST. Incubation with SOX30 primary antibodies diluted at 1:500 in blocking solution was then carried out overnight at 4°C with gentle rocking. The membrane was then washed three times with TBST before incubation with HRP conjugated anti-mouse or anti-rabbit secondary antibody at 1:2000 dilution for 1 hour. Excess secondary antibodies were removed with three TBST washes and ECL substrate (Clarity Western ECL Substrate, Bio-RAD) was applied for detection on a Li-Cor Odyssey Fc Imager.

## Immunofluorescence

Wildtype and *Sox30*-null postnatal testes embedded in paraffin were sectioned at 7µm and dewaxed by immersion into xylene twice for 10 minutes and then rehydrated through an ethanol series ranging from 100% to 35% ethanol (v/v) in water. Heat induced antigen retrieval was carried out with Antigen Unmasking Solution, Tris-Based (Vector Laboratories) and allowed to gradually cool to room temperature for approximately 1 hour. The sections were then washed twice with water, PBS and then PBTX before blocking in 10% heat-inactivated horse serum in PBTX for at least 1 hour. Sections were incubated at 4°C overnight with SOX30 primary antibodies diluted to 1:100 in blocking solution. The sections were washed twice with PBTX and incubated with anti-rabbit or anti-mouse secondary antibody conjugated to Alexa Fluor 568 (Thermo Fisher Scientific) diluted at 1:200 in blocking solution for 1 hour. Excess secondary antibodies were removed with three PBTX washes and the sections were stained with DAPI (10μg/ml) for 10 minutes before mounting in aqueous media.
